# Dr. Charles Welch

**Published:** 1900-11-15

**Authors:** 


### OBITUARY.

### DR. CHARLES WELCH.

Dr. Charles Welch, of Wilmington, O., died September 9, 1900, at his home, of heart trouble from inflammatory rheumatism. Dr. Welch was well known, not only in southern Ohio, but took an active interest in his profession and was always in attendance at the local and State meetings, and a frequent attendant at the national assemblies. He was a son of Dr. L. B. Welch, one of the early practitioners of dentistry. He was a graduate of the Ohio College of Dental Surgery in the class of 1871, and practiced in his native town for nearly thirty years. He was born July 4, 1850. He was a student and a conservative practitioner. Had many official distinctions forced upon him, as he was a modest gentleman and well thought of by all who knew him. He was president of the Ohio State Dental Association and also of the Mississippi Valley Dental Association. He was married and leaves a widow with no children. He will be greatly missed as one of the regular attendants at the State meetings, and the profession has sustained a real loss in his death.

